# Liver Transplantation Following Hepatocellular Carcinoma Rupture and Long-Term Immunotherapy: A Case Report

**DOI:** 10.1007/s12029-026-01573-0

**Published:** 2026-08-15

**Authors:** Lorenza Di Marco, Marco Scoppettuolo, Alessandra Pivetti, Nicola De Maria, Veronica Bernabucci, Antonio Colecchia, Cristian Caporali, Federico Casari, Annarita Pecchi, Andrea Spallanzani, Massimiliano Salati, Barbara Catellani, Gian Piero Guerrini, Stefano Di Sandro, Massimo Dominici, Fabrizio Di Benedetto

**Affiliations:** 1 Department of Oncology and Hematology, Oncology Unit, University Hospital of Modena and Reggio Emilia, University of Modena and Reggio Emilia, Modena, 41124 Italy; 2 Chimomo Department, Gastroenterology Unit, University Hospital of Modena and Reggio Emilia, University of Modena and Reggio Emilia, Modena, 41124 Italy; 3 Department of Radiology, University of Modena and Reggio Emilia, Modena, Italy; 4 Hepato-Pancreatic-Biliary Surgery and Liver Transplantation Unit, University Hospital of Modena and Reggio Emilia, University of Modena and Reggio Emilia, Modena, 41125 Italy; 5 Liver Surgery and Liver and Kidney Transplantation, ASST GOM Niguarda, Milan, Italy

**Keywords:** Hepatocellular Carcinoma, Liver Transplantation, Immune Checkpoint Inhibitors, Hemoperitoneum, Pexa-Vec

## Abstract

**Aim:**

To report a case of advanced hepatocellular carcinoma (HCC) complicated by tumor rupture, successfully downstaged with combined oncolytic immunotherapy (Pexa-Vec) and a PD-1 inhibitor (Nivolumab), ultimately enabling liver transplantation (LT). This report highlights the potential role of immune checkpoint inhibitors (ICIs) as bridging therapy in otherwise ineligible patients.

**Methods:**

A 54-year-old male with HCV-related cirrhosis presented with multifocal, unresectable HCC (BCLC C) complicated by hemoperitoneum. Surgery was not indicated. He was enrolled in a Phase I/IIa trial of intralesional Pexa-Vec and intravenous Nivolumab (240 mg every 14 days). Percutaneous biopsies confirmed Edmondson–Steiner grade 2 HCC. Clinical, laboratory, and imaging follow-up were performed throughout therapy. Pre-transplant screening was performed after 4 years of stable disease.

**Results:**

The patient received three intralesional Pexa-Vec injections and 93 cycles of Nivolumab over four years, with only mild, transient adverse events. Imaging showed stable disease without extrahepatic spread. Pre-LT evaluation confirmed preserved liver function (Child-Pugh A5, ECOG 0). Immunotherapy was suspended 12 weeks before LT, and LT was performed successfully in September 2023. Post-transplant follow-up (24 months) revealed no recurrence of HCC or graft rejection. HCV was eradicated with direct-acting antivirals. Explant pathology showed complete necrosis in injected nodules and regressive changes in other lesions.

**Conclusions:**

ICIs alone or in combination with oncolytic immunotherapy may serve as novel downstaging strategies in advanced, ruptured HCC. Successful LT with long-term disease-free survival challenges traditional concerns about peritoneal seeding. Larger studies are required to define optimal patient selection, timing, and safety of pre-transplant ICIs.

**Lay summary:**

A 54-year-old man with advanced, ruptured liver cancer had an unexpectedly stable course over four years while receiving immunotherapy and an oncolytic virus. He later underwent a liver transplant and remains cancer-free two years later, illustrating the unpredictable nature of some severe liver cancer cases.

**Clinical trial registration:**

Not applicable.

## Introduction

Liver transplantation (LT) provides the highest chance of long-term survival in patients with hepatocellular carcinoma (HCC) and cirrhosis, treating both the tumor and underlying liver disease [1]. Access is restricted by strict selection criteria to minimize post-transplant recurrence, excluding many patients with intermediate- or advanced-stage HCC.

Downstaging strategies aim to expand eligibility, integrating tumor burden, biology, and treatment response into decision-making [2, 3]. Systemic therapy for advanced HCC has evolved: after the era of multikinase inhibitors, immune checkpoint inhibitors (ICIs) have reshaped treatment paradigms, demonstrating survival benefits with favorable safety [4, 5].

Combination approaches are emerging. Oncolytic virotherapy, such as Pexa-Vec (JX-594), selectively infects tumor cells, induces oncolysis, activates the immune system, and exerts antiangiogenic effects [6–8]. Viral-mediated immune priming may enhance PD-1 inhibitor efficacy, providing theoretical synergy [6].

Spontaneous HCC rupture carries a poor prognosis due to a high risk of peritoneal dissemination, often precluding LT [9, 10]. The role of immunotherapy-based bridging strategies in this context is largely unexplored. We report a case of ruptured, multifocal advanced HCC successfully managed with prolonged combined immunotherapy, enabling LT without recurrence.

## Case Report

In June 2019, a 54-year-old Italian male presented to a Spanish hospital with acute abdominal pain, nausea, vomiting, diaphoresis, and hypotension. History included controlled hypertension and active smoking. CT revealed a large hemoperitoneum and multifocal liver lesions (most significant: 8 cm in segment VIII, 4 cm in segment III), consistent with advanced HCC without biliary invasion or portal vein thrombosis. Alpha-fetoprotein (AFP) was 219.8 ng/mL. Tumor rupture was presumed to be the source of bleeding.

Transferred to our tertiary center, ultrasound-guided biopsy confirmed grade 2 HCC (Edmondson–Steiner) and HCV-related cirrhosis. Given multifocal BCLC C disease, surgical or locoregional therapy was not indicated. Preserved performance status (ECOG 0) and liver function (Child-Pugh A5) allowed enrollment in a Phase I/IIa trial evaluating intralesional Pexa-Vec plus Nivolumab (240 mg IV every 14 days) as first-line therapy.

Three intralesional Pexa-Vec injections (on days 1, 15, and 29) were administered into a segment V lesion. Nivolumab started concurrently. Mild adverse events included transient right bundle branch block and minor laboratory inflammatory changes. After the start of systemic therapy, there was a drop-out of AFP, which remained constantly at values lower than 3 ng/ml. Therapy continued post-trial closure until March 2024 (93 infusions).

**Fig. 1:**
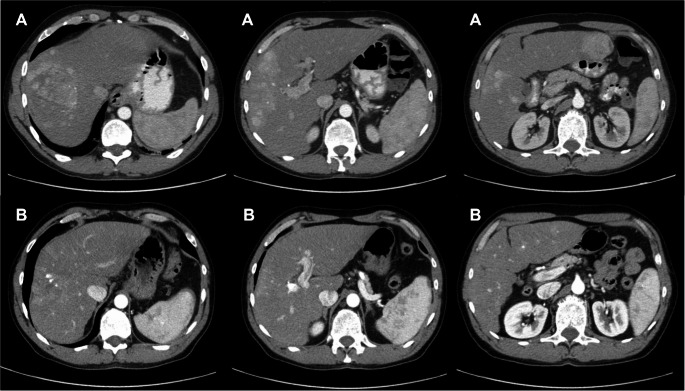
A: CT scan at baseline. B: CT scan after 93^h^ of Nivolumab infusion

Serial imaging demonstrated stable disease without extrahepatic spread. In 2023, after nearly 4 years of disease control, a multidisciplinary evaluation found LT feasible. Immunotherapy was discontinued in May 2023, and LT was performed in September 2023, 12 weeks later (Fig. 1).

### Explant Pathology

Histology confirmed cirrhosis and chronic HCV infection. The Pexa-Vec–injected lesion showed complete necrosis and fibrosis, with no viable tumor. Other nodules showed regressive changes; microvascular invasion was absent. No peritoneal seeding or extrahepatic disease was observed.

**Fig. 2:**
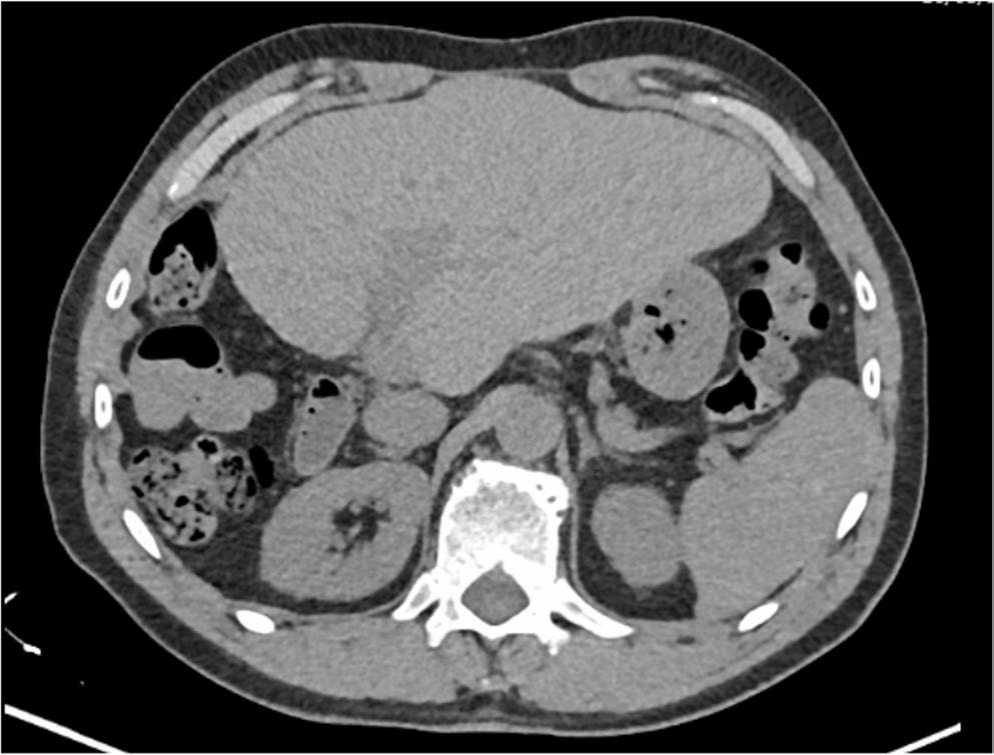
Last follow-up abdominal CTscan after Liver Transplant (May 2026)

**Fig. 3:**
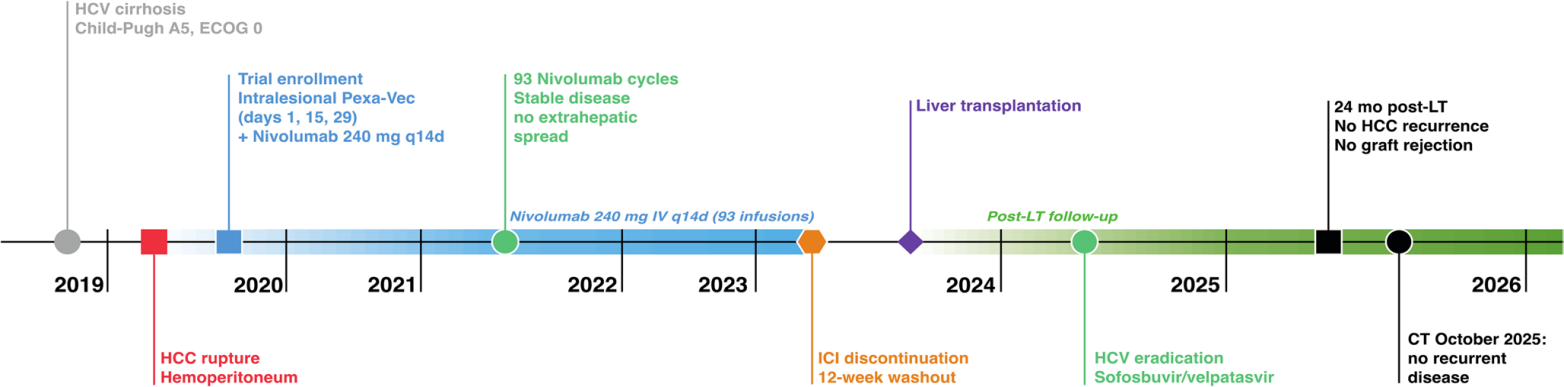
Timeline of clinical history

At 24 months post-LT, the patient remains well, without HCC recurrence or graft rejection. HCV was eradicated with sofosbuvir/velpatasvir. The most recent CT (May 2026) confirmed the absence of recurrent disease (Figs. 2 and 3).

## Discussion

This case illustrates exceptional long-term disease control in advanced, ruptured, multifocal HCC, which has historically been considered ineligible for LT [1, 9]. Systemic therapy in this setting was traditionally palliative. ICIs now enable durable responses in selected patients [4, 5].

The prolonged stability of Nivolumab over 4 years demonstrates that extended exposure is feasible with careful monitoring. Durable responses may enable systemic therapy to serve as a biologic selection tool for LT, aligning with multiparametric treatment strategies [2, 3].

While the contribution of Pexa-Vec is uncertain, explant pathology supports a local effect. Pexa-Vec induces tumor lysis, immune activation, and antiangiogenic activity, potentially synergizing with PD-1 blockade [6–8].

Ruptured HCC usually precludes LT because of the risk of peritoneal seeding [9, 10]. The absence of peritoneal disease in this case suggests that systemic immune control may mitigate spread, warranting further investigation.

Pre-LT ICIs raise concerns about allograft rejection. Recent systematic reviews and meta-analyses suggest that, with appropriate washout, pre-LT ICIs may be feasible [11–13].

Ethical considerations were addressed through trial approval, institutional oversight, and evaluation by a multidisciplinary transplant committee. LT was performed only after prolonged disease stability and ICI discontinuation to minimize the risk of rejection.

## Conclusion

We report a patient with ruptured, multifocal, advanced HCC who achieved prolonged disease control with combined immunotherapy and underwent LT without recurrence. Although generalization is limited, this case supports further study of ICIs, alone or with oncolytic virotherapy, as downstaging strategies for highly selected patients.

## Data Availability

No datasets were generated or analysed during the current study.

## Abbreviations

HCC: Hepatocellular Carcinoma
AFP: Alpha-fetoprotein
CT: Computed Tomography
DAAs: Direct-Acting Antivirals
ECOG-PS: Eastern Cooperative Oncology Group
ICIs: Immune Checkpoint Inhibitors
LT: Liver Transplantation
PCR: C-Reactive Protein
PD-1: Programmed Cell Death Protein
PD-L1: Programmed Death-Ligand 1
Pexa-Vec: Pexastimogene Devacirepvec
TKI: Tyrosine Kinase Inhibitor
